# Exploring the 2D and 3D structural properties of topologically associating domains

**DOI:** 10.1186/s12859-019-3083-z

**Published:** 2019-12-02

**Authors:** Tong Liu, Zheng Wang

**Affiliations:** Department of Computer Science, University of Miami, 1365 Memorial Drive, P.O. Box 248154, Coral Gables, FL 33124 USA

**Keywords:** Hi-C, Chromosomal three-dimensional structure, Topologically associating domains, 3D structural properties of TADs

## Abstract

**Background:**

Topologically associating domains (TADs) are genomic regions with varying lengths. The interactions within TADs are more frequent than those between different TADs. TADs or sub-TADs are considered the structural and functional units of the mammalian genomes. Although TADs are important for understanding how genomes function, we have limited knowledge about their 3D structural properties.

**Results:**

In this study, we designed and benchmarked three metrics for capturing the three-dimensional and two-dimensional structural signatures of TADs, which can help better understand TADs’ structural properties and the relationships between structural properties and genetic and epigenetic features. The first metric for capturing 3D structural properties is radius of gyration, which in this study is used to measure the spatial compactness of TADs. The mass value of each DNA bead in a 3D structure is novelly defined as one or more genetic or epigenetic feature(s). The second metric is folding degree. The last metric is exponent parameter, which is used to capture the 2D structural properties based on TADs’ Hi-C contact matrices. In general, we observed significant correlations between the three metrics and the genetic and epigenetic features. We made the same observations when using H3K4me3, transcription start sites, and RNA polymerase II to represent the mass value in the modified radius-of-gyration metric. Moreover, we have found that the TADs in the clusters of depleted chromatin states apparently correspond to smaller exponent parameters and larger radius of gyrations. In addition, a new objective function of multidimensional scaling for modelling chromatin or TADs 3D structures was designed and benchmarked, which can handle the DNA bead-pairs with zero Hi-C contact values.

**Conclusions:**

The web server for reconstructing chromatin 3D structures using multiple different objective functions and the related source code are publicly available at http://dna.cs.miami.edu/3DChrom/.

## Background

Revealing the three-dimensional (3D) structures of chromosomes has been an important and challenging research topic. Fluorescence in situ hybridization (FISH) can measure the presence of specific DNA sequences, but provides limited spatial distance information; therefore, we cannot massively model chromatin 3D structures using FISH data. In comparison, the chromosome conformation capture (3C) technique [1] can capture physical interactions between two genome loci; the chromosome conformation capture-on-chip (4C) technique [2, 3] can capture the interactions between one genomic site and any genomic sites; and the chromosome conformation capture carbon-copy (5C) technique [4] can detect chromatin interactions among a set of genomic loci. Particularly, the recent population-cell Hi-C [5] technique, which can capture chromatin interactions across the entire genome, provides us an opportunity to more accurately explore the relationships between the spatial organizations of genomes and genomic functions [6–9]. A population-cell Hi-C contact profile usually is generated from millions of cells.

Various computational methods using Hi-C data as input to infer chromatin 3D structures have been developed. Duan et al. [10] firstly introduced metric multidimensional scaling (MDS) for reconstructing chromatin 3D structures. After that, Varoquaux et al. [11] and Ay et al. [9] designed new objective functions of metric MDS to model the 3D structures of chromatins based on experimental and simulated Hi-C data. Hu et al. [8] developed a Bayesian approach (BACH) based on an assumption that Hi-C contact counts follow a Poisson distribution. After removing systematic biases in the Hi-C data, they used Markov chain Monte Carlo simulations to infer chromosomal 3D structures. Zhang et al. developed ChromSDE [12] that infers 3D structures of chromosomes by semi-definite programming and uses golden section search to find the best parameter for converting Hi-C contacts into spatial target distances. While the above-mentioned studies are based on population-cell Hi-C data, it is important to notice the emergence of single-cell Hi-C technique revealing cell-to-cell variabilities and related method that can reconstruct chromosomal 3D structures based on single-cell Hi-C data [13, 14].

Topologically associating domains (TADs) have been identified as self-interacting genome regions detected from the normalized Hi-C contact matrices [7]. The Hi-C contacts within TADs are apparently larger and more enriched than those between two successive TADs. Some factors are enriched at the boundary regions of TADs [7] including RNA polymerase II, insulator binding protein CTCF, and H3K4me3. It is also found that TADs are conserved across different cells or cell lines and even among different species, indicating that TADs may be the structural units of genomes [7].

Hu et al. [8] defined a structural metric named HD ratio to quantify TAD structures’ elongation properties. They found that the elongated TADs usually had a large value of HD ratio. They also observed that HD ratios were positively correlated with selected genetic and epigenetic features [8]. However, the limitation of HD ratio is that it can only measure the elongation properties but not the compactness or folding degree in the 3D space, both of which are significant structural properties.

We benchmarked three measures to gauge TADs’ structural properties, which are based on radius of gyration [15], folding degree [16], and exponent parameter [5]. We have observed that the structural properties of TADs are highly correlated with multiple genetic and epigenetic features. We observed that small values of radius of gyration and folding degree usually correspond to a strong enrichment of CTCF, which plays an important role in organizing the 3D structures of chromosomes [17], whereas the exponent parameter usually has a significant positive correlation with the intensities of CTCF. The 3D structures of TAD used in this study were generated by multidimensional scaling based on population-cell Hi-C data. We benchmarked and compared four objective functions for reconstructing chromosomal 3D structures including an objective function newly designed to model the cases with zero Hi-C contact value.

## Methods

### Resource and processing of Hi-C data, TAD definitions, and chromatin states information

We downloaded the Hi-C contact matrices at 40 kb resolution from http://chromosome.sdsc.edu/mouse/hi-c/download.html, which includes raw and normalized Hi-C matrices of each chromosome for mouse embryonic stem cells (mESC). The domaincaller software [7] was used to detect TADs on normalized Hi-C contact matrices at 40 kb resolution, resulting in 2040 TADs. The 3D structures of these 2040 TADs were reconstructed and used to explore the relationships between 3D DNA structures and genetic and epigenetic features.

We downloaded the low-resolution raw Hi-C reads from the GEO database with ID GSE35156 [7]. The low resolutions we used in our analysis are 400 kb, 500 kb, and 1 Mb. BWA [18] with default parameters was used to separately map paired-end Hi-C reads back to the mouse reference genome (build mm9). We removed multiple experimental artifacts defined in [19]: first, the PCR duplicates, resulting in multiple paired-end reads mapped to the same genomic location were removed using Picard (http://broadinstitute.github.io/picard/); second, we discarded the reads with mapping quality less than or equal to 10; and third, paired-end reads were discarded if the two ends were mapped to the same fragment (i.e., a genomic region defined by two successive restriction sites) or the sum of the distances between the mapping site of each end of a paired-end read and its corresponding nearest restriction cut site was larger than a certain threshold (500 bp suggested in [6]). We used the ICE normalization method [20] to normalize the raw Hi-C contact matrices at resolutions of 400 kb, 500 kb, and 1 Mb.

We downloaded the 14-state annotations for mESC from [21]. These chromatin states were generated using ChromHMM [22]. For each TAD, we computed its fold enrichment of each state using the OverlapEnrichment function in ChromHMM [22]. For each pair of TADs, we calculated the absolute value of the Pearson’s correlation coefficient between their fold enrichments, which was used as their similarity scores. We used Spectral Clustering [23, 24] with predefined number of clusters equal to 20 to classify TADs based on their chromatin-state similarity scores.

### Data for genetic and epigenetic features

We defined the gene density of a TAD as the number of transcription start sites (TSS) located within the TAD. The number of peaks existing in a TAD was used as the enrichment for both H3K4me3 (GEO, GSM723017) and RNA polymerase II (GEO, GSM723019). The enrichment for the other four features including CTCF (GEO, GSM723015), H3K27me3 (GEO, GSM1000089), H3K9me3 (GEO, GSM1000147), and H3K36me3 (GEO, GSM1000109), were defined as the average (after log2 scale) of all data values existing in a TAD. All enriched values for a TAD were normalized by the TAD's length.

### Reconstructing the 3D structures of chromosomes and TADs using metric MDS

The Hi-C contact data were formulated as a contact matrix as in [5]. The DNA of the whole chromosome or TAD was evenly split into beads/bins with length equal to the resolution value. The entry *c*_*ij*_ in a Hi-C contact matrix *C* denotes the contact count between the *i*^*th*^ and *j*^*th*^ beads. All entries in the matrix *C* can be simply classified into two mutually exclusive sets: *C*_1_ = {(*i*, *j*)| *c*_*ij*_ ≠ 0} and *C*_2_ = {(*i*, *j*)| *c*_*ij*_ = 0}, that is, the first set *C*_*1*_ contains the bead pairs with the number of Hi-C contact larger than zero and the second set *C*_*2*_ contains the bead pairs having zero Hi-C contact.

To model the 3D structures of TADs and individual chromosomes by metric MDS, the Hi-C contact values between any two DNA beads are converted into a target spatial distance. The conversion was conducted using the following equation:

1 $$ {\delta}_{ij}=\beta {\left(\raisebox{1ex}{$1$}\!\left/ \!\raisebox{-1ex}{${c}_{ij}$}\right.\right)}^{\alpha }\  if\ \left(i,j\right)\in {C}_1, $$

 where α was set to 1/3 as in [11]; and β, the scale parameter [11], was set to 1.

We implemented and benchmarked the following three objective functions:

2 $$ {\min}_{x,y,z}{\sum}_{\left(i,j\right)\in {C}_1}\frac{{\left({d}_{ij}-{\delta}_{ij}\right)}^2}{{\delta_{ij}}^2} $$

3 $$ {\min}_{x,y,z}{\sum}_{\left(i,j\right)\in {C}_1}\frac{{\left({d}_{ij}-{\delta}_{ij}\right)}^2}{\delta_{ij}}-\gamma {\sum}_{\left(i,j\right)\in {C}_2}{d_{ij}}^2 $$

4 $$ {\min}_{x,y,z}{\sum}_{\left(i,j\right)\in {C}_1}\frac{{\left({d}_{ij}-{\delta}_{ij}\right)}^2}{{\delta_{ij}}^2}+{\sum}_{\left(i,j\right)\in {C}_2}\frac{{\left({d}_{ij}-R\right)}^2}{R^2} $$

The *d*_*ij*_ and δ_ij_ are the real distance and target/objective distance between beads *i* and *j*, respectively. The first objective function [25] is from [11], whose numerator term is normalized by δ_ij_^2^.

The second one is from [12], in which the second term is a regularization term for maximizing the pairwise distances between two beads in the set *C*_*2*_. The parameter γ in the second term was set to 0.01 [12].

We designed the third objective function based on the first two functions by introducing the second term, which was used to make the distances between any two beads in *C*_*2*_ as close to value *R* as possible. We tested two different ways of defining *R*: (1) as the sum of the edge weights along the shortest path found by the Floyd-Warshall algorithm [26] if we think of every DNA bead as a node in a graph and that every two DNA beads have an edge if they have >0 Hi-C contacts; the weight of each edge is modeled as the target distance; and (2) as the maximum target distance between any two bead pairs in *C*_*1*_.

### Radius of gyration

The reconstructed 3D structure of a TAD is represented using a n-by-3 matrix where n is the number of beads in a TAD and 3 indicates the 3D coordinates.

The centre of mass *R*_*C*_ of a TAD 3D structure is calculated by:

5 $$ {\sum}_{i=1}^n{m}_i\left({x}_i-{R}_C\right)=0, $$

 where *m*_*i*_ is the mass of the *i*^*th*^ bead and *x*_*i*_ is the 3D coordinates of the bead.

The radius of gyration *R*_*g*_ of a TAD is calculated as:

6 $$ {R}_g={\left({\sum}_{i=1}^n{m}_i{\left({x}_i-{R}_C\right)}^2/M\right)}^{\frac{1}{2}}, $$

 where *M* is all beads’ mass values normalized by the TAD length.

**Fig. 1:**
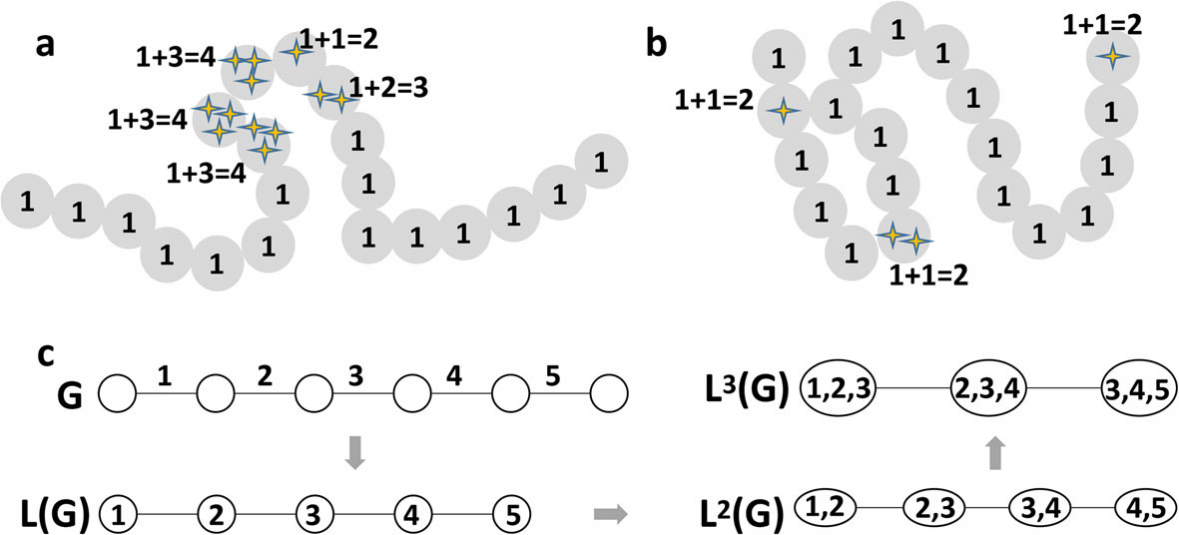
An illustration of radius of gyration. **a** and **b** illustrate two TADs with the same size but different 3D structures. The stars indicate TSS. **c** An example of the first, second, and third line graphs of a given graph G. G is the original chain of beads, in which every two adjacent beads have an edge. L(G) is the first line graph, in which each node represents an edge in G. An edge is created in L(G) between two nodes if these two nodes represent two sequentially adjacent edges in G. In the same way, the second and third line graphs can be generated

We first assumed the mass values of all beads in one TAD were the same. The unit value 1 was used to represent the mass of every bead, which makes the radius of gyration to only consider the spatial arrangement of nucleotide. This type of mass representation is labelled as “mass_unit” in this paper. We also considered gene density that is, the number of transcription start sites (TSS) as the mass representations. Figure 1 (a) and (b) show two TADs with the same length but with different 3D structures, which have different distributions of TSS. The 3D structure shown in Fig. 1(a) is dispersed but with four successive beads enriched with TSS concentrated in a small region of the TAD, whereas the structure shown in Fig. 1(b) is compacted but with three beads having one or two TSS spreading over the TAD. The illustration in Fig. 1(a) and (b) indicates that the compactness of all DNA beads can be different compared with the compactness of the DNA beads with a certain genetic feature. Therefore, the second mass representation “mass_TSS” was modeled as the number of TSS located within a bead plus the unit mass.

We defined another two mass representations “mass_or” and “mass_and”. “mass_or” defines the mass of a bead as 2 if we find one of the three features (H3K4me3, RNA polII, and TSS) in the bead, and 1 if we find none of them. “mass_and” defines the mass of a bead as the sum of 1 (indicating nucleotide), the total number of TSS, and the number of peaks for H3K4me3 and RNA polII.

### Folding degree and exponent parameter

The Estrada index [16, 27] has been used as an effective metric for measuring the folding degree of protein tertiary structures. In this study, we used it to model the folding degree of the 3D structures of TADs. Every three consecutive DNA beads in a chain of beads (e.g., a TAD) can form a plane; and every two consecutive planes have a specific dihedral angle. The Estrada index is able to capture the folding degree of the entire chain while considering these dihedral angels. It generates the first, second, and third line graphs of a given graph *G* (Fig. 3(c)) and calculates the eigenvalues of a symmetric matrix from the third line graph to obtain the final Estrada index.

We also applied and benchmarked a two-dimensional (based on Hi-C contact matrix) structural metric named exponent parameter [5].

### Normalized Hi-C data between TADs with different lengths

Since the lengths of TADs are different, the Hi-C interaction frequencies between the Xist-containing TAD (the TAD including the Xist gene) and each of the other TADs were calculated as the observed/expected numbers of Hi-C contact counts (in this way, the Hi-C contacts were normalized by the different lengths of TADs): as previously did in [5, 28], the expected contacts between two different TADs *i* and *j* were calculated by *E*_*ij*_ *= R*_*i*_ *× R*_*j*_ *× N*_*inter*_, where *R*_*i*_ and *R*_*j*_ are the fractions of inter-TADs reads associated with *i* and *j* respectively, and *N*_*inter*_ is the total number of inter-TADs reads.

## Results

### Structural properties are correlated with genetic and epigenetic features

We calculated the Pearson’s correlation between each of the three measures, including radius of gyration, folding degree, and exponent parameter, and each of the seven features, including gene density, H3K36me3, H3K9me3, H3K27me3, H3K4me3, RNA polII, and CTCF. The results are shown in Table 1.

**Table 1:** The Pearson’s correlation coefficients between the three measures of 3D and 2D structural properties and genetic/epigenetic features

Methods	Gene density	CTCF	RNA polII	H3K4me3	H3K9me3	H3K27me3	H3K36me3
Folding degree	−0.135	− 0.133	− 0.189	−0.164	− 0.003	−0.009	− 0.157
Rg (mass_unit)	−0.198	− 0.460	−0.304	− 0.266	−0.410	− 0.405	−0.447
Rg (mass_TSS)	−0.229	−0.467	− 0.319	−0.279	− 0.404	−0.402	− 0.455
Rg (mass_or)	−0.205	− 0.455	−0.311	− 0.272	−0.396	− 0.394	−0.447
Rg (mass_and)	−0.218	−0.457	− 0.311	−0.271	− 0.395	−0.395	− 0.449
Exponent parameter	0.298	0.491	0.370	0.423	0.313	0.355	0.500

**Fig. 2:**
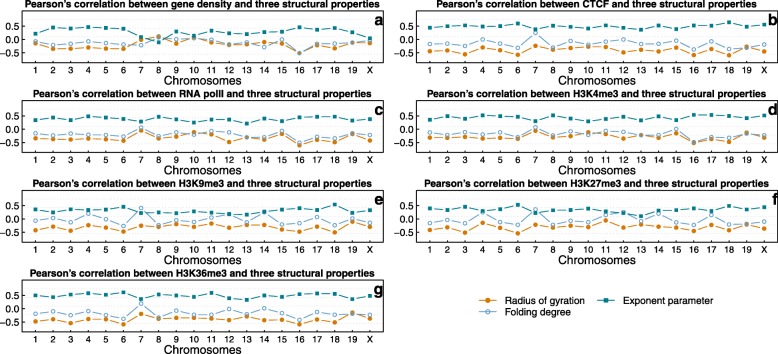
The Pearson’s correlation coefficients between the three measures (radius of gyration, folding degree, and exponent parameter) and seven features: (**a**) gene density, (**b**) CTCF, (**c**) RNA polII, (**d**) H3K4me3, (**e**) H3K9me3, (**f**) H3K27me3, and (**g**) H3K36me3

We observed that the radius of gyration is negatively correlated with the seven features; folding degree is also negatively correlation with the seven features, but not as significant as radius of gyration. However, exponent parameter of TADs’ 2D contact matrices has strong positive correlations with these features. All of the correlation values shown in Table 1 are statistically significant (all *P*-values < 10^− 5^). We may conclude from the significant negative correlations between radius of gyration and CTCF that the compactness level of TADs in the 3D space are related to the enrichment level of CTCF. The Pearson’s correlations between the three measures and the seven features on each chromosome are shown in Fig. 2.

### Relationships between structural properties and chromatin states

**Fig. 3:**
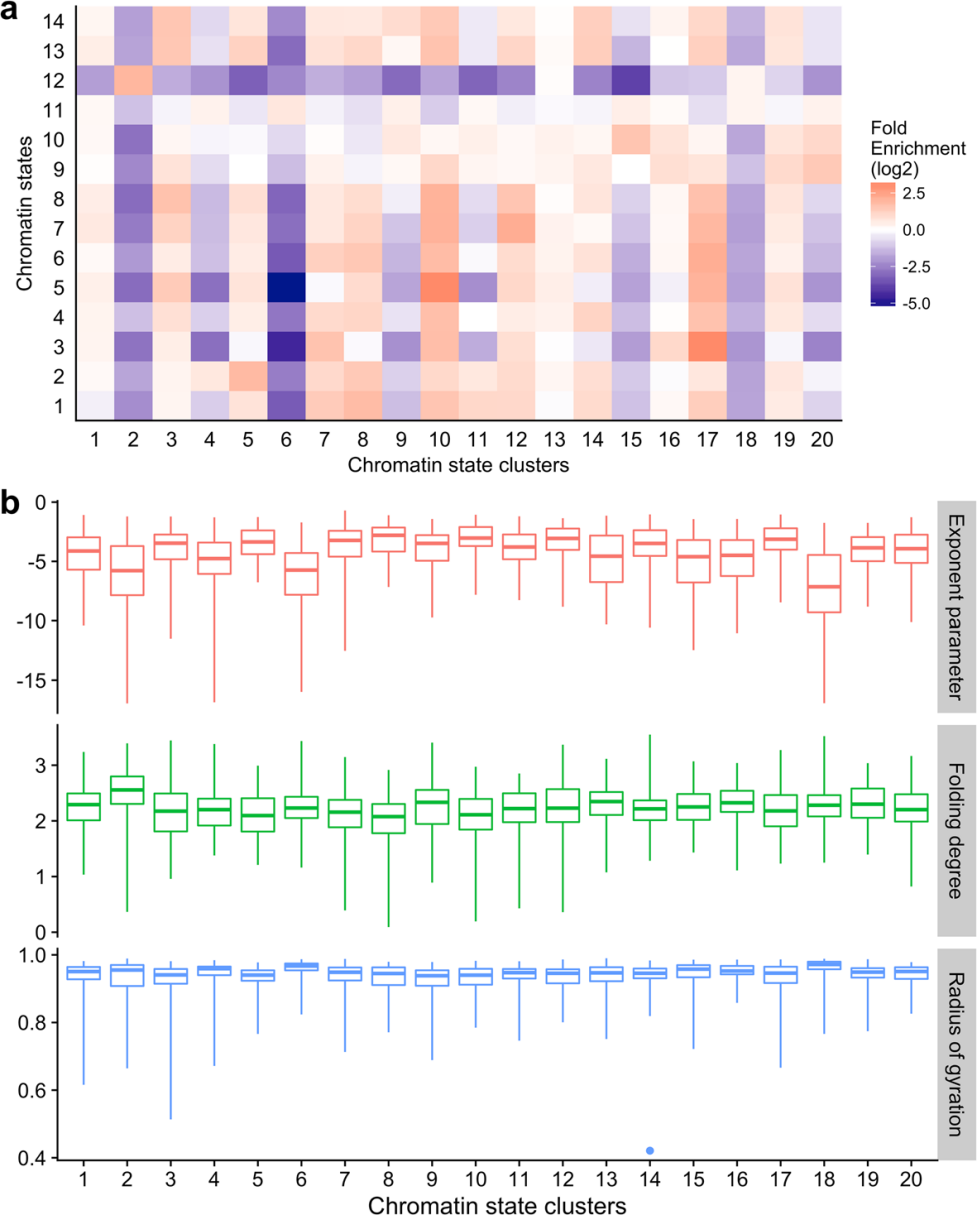
**a** Fold enrichment of the 20 chromatin state clusters (log2 scale). **b** The boxplots of exponent parameter, folding degree, and radius of gyration for TADs in each of the 20 chromatin state clusters

The 14 chromatin states we have considered in this study include: (1) transcription elongation, (2) weakly transcribed, (3) weak/poised enhancer 1, (4) active promoter 1, (5) strong enhancer 1, (6) active promoter 2, (7) strong enhancer 2, (8) weak/poised enhancer 2, (9) poised promoter, (10) repressed, (11) heterochromatin 1, (12) heterochromatin 2, (13) heterochromatin 3, and (14) insulator. We clustered all 2040 TADs based on their chromatin-state enrichment similarities (details see Methods). The clustering procedure generated 20 clusters of TADs. Based on the fold enrichment of each chromatin state for each of the 20 clusters (Fig. 3 (a)), we found that: (1) three clusters (i.e., clusters 2, 6, and 18) are depleted for almost all of the 14 chromatin states; (2) compared with the TADs in the other clusters, the TADs in the same three clusters have apparently smaller exponent parameters and larger radius of gyrations (Fig. 3 (b)).

### Inference and evaluations of the reconstructed 3D structures

#### Evaluations of the objective functions

Three different objective functions were used to reconstruct the 3D structures of 20 chromosomes of mES individually at two different resolutions (1 Mb and 500 kb).

**Fig. 4:**
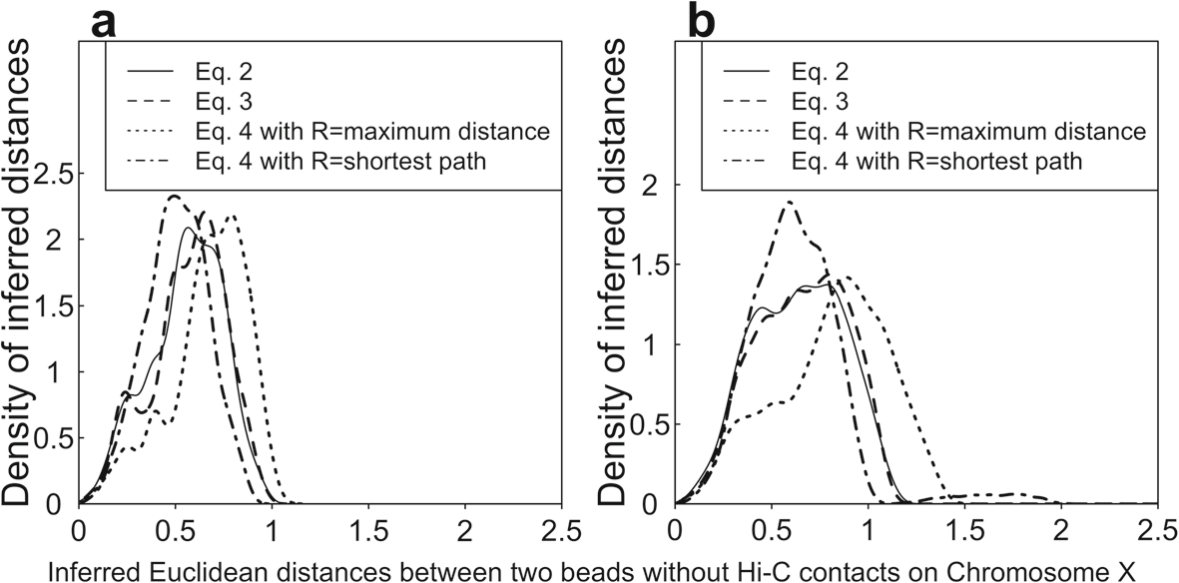
The distribution of inferred Euclidean distances between any two beads with zero Hi-C contacts at 1 Mb (**a**) and 500 kb (**b**) resolutions

We plotted the distribution of the inferred Euclidean distances between any two beads with missing Hi-C contacts on the X-chromosome at 1 Mb and 500 kb resolutions in Fig. 4. Compared to the other objective functions, Eq. (4) with *R* equal to the maximum distance generates larger distances. Therefore, our Eq. 4 with *R* equal to the maximum distance works best if we assume that the two beads with missing Hi-C contacts should be spatially far away to each other. Our results shown in Fig. 4 indicates that Eq. (4) with *R* equal to the shortest path generates smaller Euclidean distances; and most of the Euclidean distances are smaller than one.

**Fig. 5:**
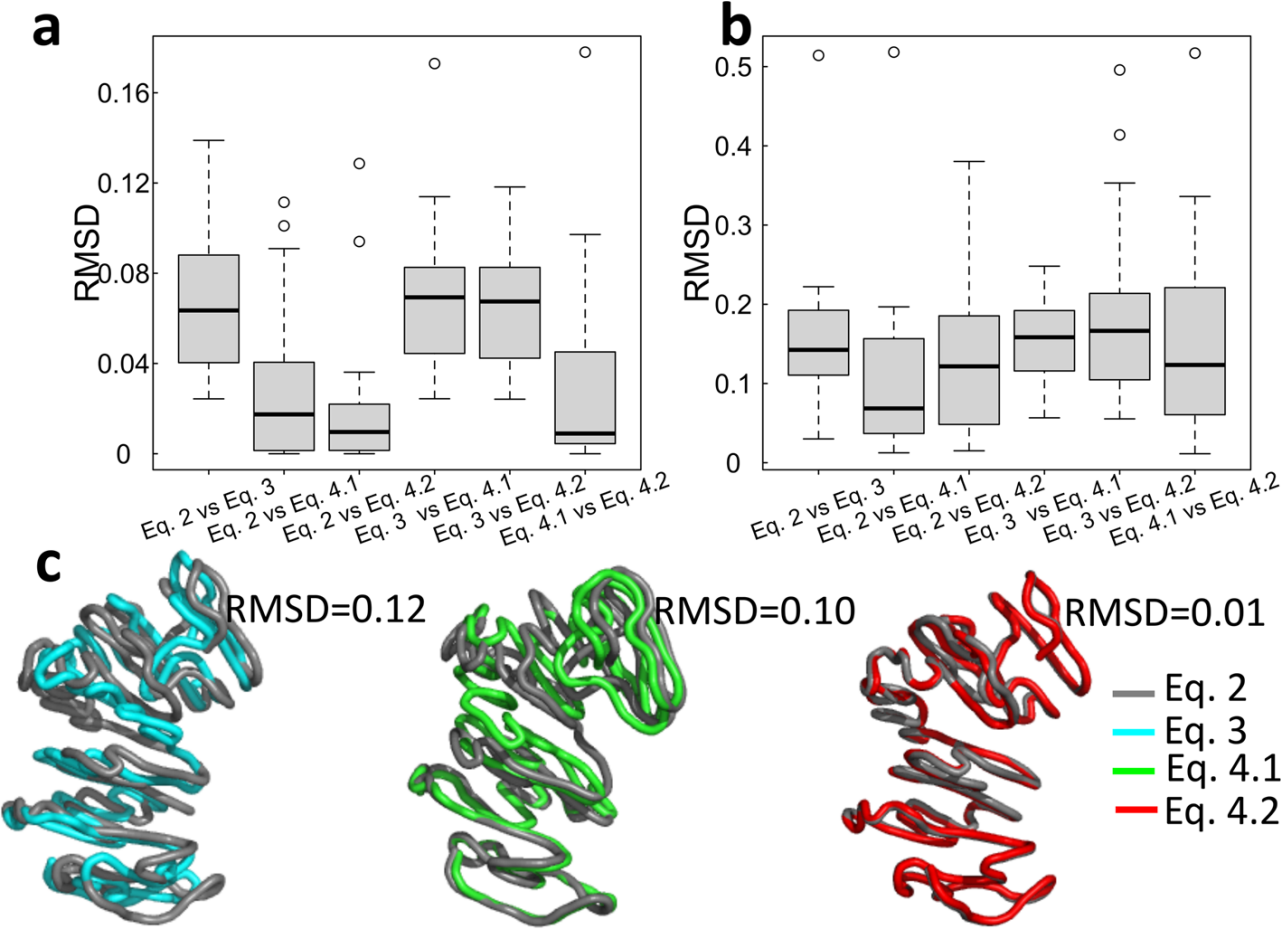
The distribution of RMSDs between any two reconstructed 3D structures inferred by the three different objective functions at 1 Mb (**a**) and 500 kb (**b**) resolutions. **c** smaller RMSD values indicate more similar structures on chromosome 8 at 1 Mb resolution

We used the Kabsch algorithm [29] to superimpose the reconstructed 3D structures from different objective functions; their minimum root-mean-square deviations (RMSDs) were calculated to quantify the level of similarity, which are shown in Fig. 5 at two different resolutions (1 Mb and 500 kb). Compared to 1 Mb resolution, the values of RMSD are relatively larger at 500 kb resolution. An illustration of the superimposed 3D structures of chromosome 8 are shown in Fig. 5(c).

**Fig. 6:**
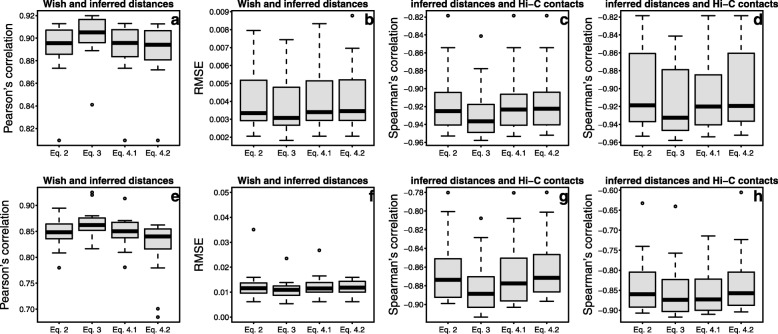
**a** and **e** show the Pearson’s correlations between the target and inferred Euclidean distances between any two beads with non-zero Hi-C contacts; **b** and **f** show the plots of root-mean-square errors (RMSEs) between wish and inferred Euclidean distances between any two beads with non-zero Hi-C contacts; **c** and **g** show the Spearman’s correlations between inferred Euclidean distances and non-zero Hi-C contact counts, whereas **d** and **h** show the same correlations as in **c** and **g** but with all Hi-C contacts. Eq. 4.1 denotes Eq. 4 with *R* equal to the maximum distance; Eq. 4.2 stands for Eq. 4 with *R* equal to the shortest path

We show more evaluation results in Fig. 6 at two different resolutions (a, b, c, d for 1 Mb and e, f, g, h for 500 kb) from different perspectives: (a) and (e) show the Pearson’s correlations between target and inferred Euclidean distances among any two beads with non-zero Hi-C contacts; (b) and (f) show the plots of root-mean-square errors (RMSEs) between target and inferred Euclidean distances among any two beads with non-zero Hi-C contacts; (c) and (g) show the Spearman’s correlations between inferred Euclidean distances and non-zero Hi-C contact counts, whereas (d) and (h) show the same correlations as in (c) and (g) but with all Hi-C contacts. These results indicate that the inferred distances with the three objective functions are consistent with Hi-C contacts; and Eq. (4) with *R* equal to the maximum distance generates slightly better results than *R* equal to the shortest path. Therefore, we used the 3D structures inferred by Eq. (4) with *R* equal to the maximum target distance in the other sections.

#### Chromatin 3D structure reconstruction and biochemistry validations with Xist lncRNA localization

**Fig. 7:**
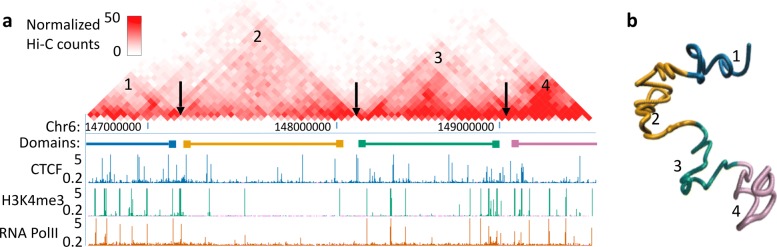
**a** An illustration of four TADs and their boundaries. **b** The reconstructed 3D structures of the four TADs were modelled at 40 kb resolution. The four TADs (i.e., 1, 2, 3, and 4) are colored by dark blue, gold, dark green, and lavender, respectively

We used metric MDS to reconstruct the 3D structures of TADs and the X-chromosome at two different resolutions. A two-dimensional heat map of Hi-C contact matrix and the corresponding reconstructed 3D structures of four TADs on chromosome 6 in mESC are shown in Fig. 7 (a, b). The 3D structures of the four TADs we inferred using Eq. (4), illustrated by four different colors, do not overlap with each other but have their own territories in the 3D space, which fits the clear boundary-defined patterns in the 2D Hi-C contact map in Fig. 7 (a).

**Fig. 8:**
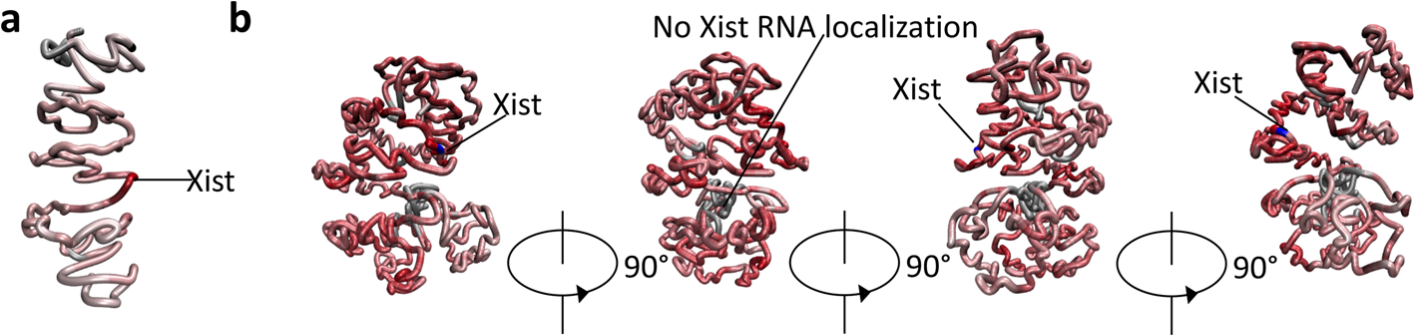
The reconstructed 3D structures of the X-chromosome with Xist transcript localization intensity mapped at 1 Mb (**a**) and 400 kb (**b**) resolutions

We also modelled the 3D structures of the X-chromosome at 1 Mb (Fig. 8 (a)) and 400 kb (Fig. 8 (b)) resolutions and further used Xist localization intensities to evaluate the inferred 3D structures. The reconstructed 3D structure of the X-chromosome at 400 kb resolution mapped with the Xist localization intensities after six hours of induction are shown in Fig. 8(b). It has been found in [30] that Xist transcripts more intensively localize in the DNA sites in spatial proximity to the Xist locus based on a high correlation (Pearson’s correlation *r* = 0.69) between Xist localization and Hi-C contact counts. If the 3D structure we inferred makes sense, a similar observation should be found.

**Fig. 9:**
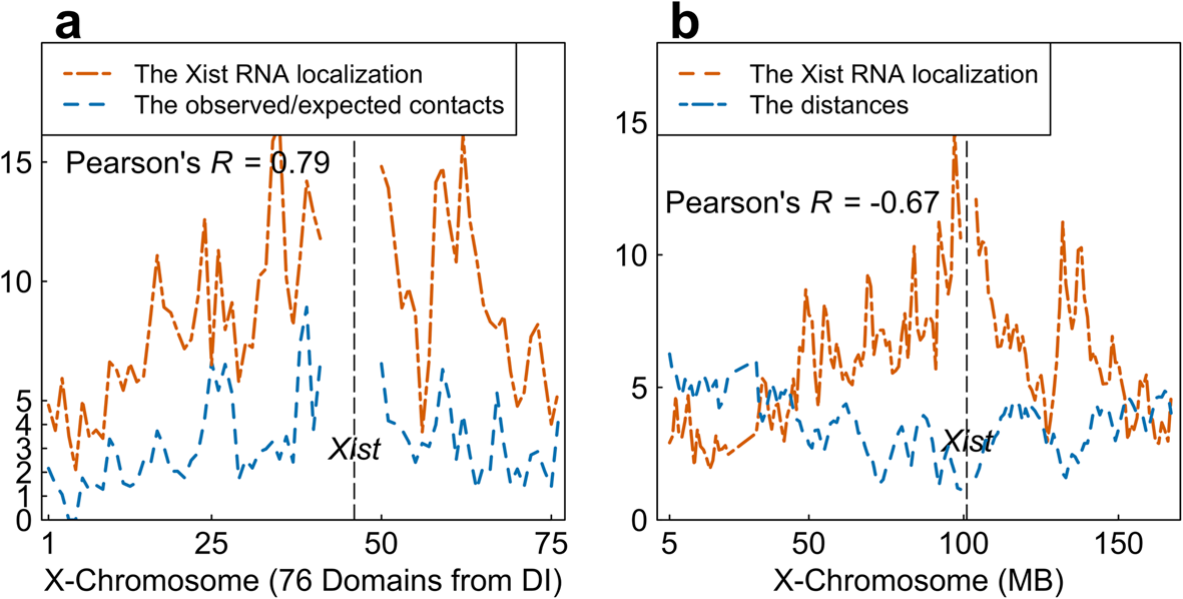
**a** The Pearson’s correlation between Xist RNA localization and observed/expected contacts. **b** The Pearson’s correlation between Xist localization degree (one hour after the start of Xist expression) and the inferred distances between each of the 1 Mb beads and the Xist-containing bead

We investigated this from the perspective of TADs (instead of DNA segments with the same length as did in [30]). We calculated the normalized Hi-C contacts between the Xist-containing TAD and all other TADs (details see Methods). The Pearson’s correlation between the localization intensities of Xist transcripts (after one hour of the induction of Xist transcription [30]) and the normalized TAD-TAD Hi-C contacts is 0.79, observed from 76 TADs in the X-chromosome and based on our inferred 3D structure of the X-chromosome (Fig. 9 (a)). This finding fits the observation mentioned in [30], indicating the correctness of our inferred 3D structure of the X-chromosome.

Furthermore, we calculated the correlation between the localization intensities of Xist transcripts and the bead-bead (the length of every bead is the same as the resolution value) spatial distances calculated based on our inferred 3D structure of the X-chromosome. First, we reconstructed the 3D structure of the X-chromosome at 1 Mb resolution (Fig. 8 (a)) and then mapped the Xist localization intensities onto the 3D structure. The Euclidean distances from the Xist-containing bead to each of the other beads were calculated, which were then correlated with the Xist transcripts localization intensities (RAP values after one hour of the induction of Xist transcription). The Pearson’s correlation is *r* = − 0.67, see Fig. 9 (b), which is consistent with what the previous work has found [30]. 

## Conclusions

In this study, we reconstructed the 3D structures of individual TADs and chromosomes of mESC at different resolutions using MDS methods and defined a new objective function to handle the cases with missing Hi-C contact.

We used three measures including exponent parameter, radius of gyration, and Estrada index to gauge the compactness and folding degree of the 3D structures of TADs and observed that the three measures are significantly correlated with seven genetic and epigenetic features including H3K36me3, H3K4me3, H3K27me3, H3K9me3, RNA PolII, gene density, and CTCF. Moreover, our novel radius-of-gyration-based measure can consider the enrichments of genetic and epigenetic features and achieve more significant negative correlation when the enrichment levels of gene density, H3K4me3, and RNA polII are in consideration. By analysing the chromatin states of TADs and conducting clustering of TADs based on chromatin states, we found that the TADs in the clusters of depleted chromatin states correspond to smaller exponent parameters and larger radius of gyration values.

In conclusion, TADs do have structural properties that significantly correlate with genetic and epigenetic features. The radius-of-gyration-based structural measure we designed can capture the structural properties of TADs and have a significant correlation with multiple genetic and epigenetic features.

## Data Availability

The web server of 3DChrom and source code are available at http://dna.cs.miami.edu/3DChrom/.

## Abbreviations

2D: Two dimensional
3D: Three dimensional
MDS: Multidimensional scaling
TAD: Topologically associating domain
